# Guidelines for Exposure Assessment in Health Risk Studies Following a Nuclear Reactor Accident

**DOI:** 10.1289/ehp.1307120

**Published:** 2013-11-01

**Authors:** André Bouville, Martha S. Linet, Maureen Hatch, Kiyohiko Mabuchi, Steven L. Simon

**Affiliations:** 1 National Cancer Institute (retired), National Institutes of Health (NIH), Department of Health and Human Services (DHHS), Rockville, Maryland, USA; 2 Division of Cancer Epidemiology and Genetics, National Cancer Institute, NIH, DHHS, Rockville, Maryland, USA

## Abstract

Background: Worldwide concerns regarding health effects after the Chernobyl and Fukushima nuclear power plant accidents indicate a clear need to identify short- and long-term health impacts that might result from accidents in the future. Fundamental to addressing this problem are reliable and accurate radiation dose estimates for the affected populations. The available guidance for activities following nuclear accidents is limited with regard to strategies for dose assessment in health risk studies.

Objectives: Here we propose a comprehensive systematic approach to estimating radiation doses for the evaluation of health risks resulting from a nuclear power plant accident, reflected in a set of seven guidelines.

Discussion: Four major nuclear reactor accidents have occurred during the history of nuclear power production. The circumstances leading to these accidents were varied, as were the magnitude of the releases of radioactive materials, the pathways by which persons were exposed, the data collected afterward, and the lifestyle factors and dietary consumption that played an important role in the associated radiation exposure of the affected populations. Accidents involving nuclear reactors may occur in the future under a variety of conditions. The guidelines we recommend here are intended to facilitate obtaining reliable dose estimations for a range of different exposure conditions. We recognize that full implementation of the proposed approach may not always be feasible because of other priorities during the nuclear accident emergency and because of limited resources in manpower and equipment.

Conclusions: The proposed approach can serve as a basis to optimize the value of radiation dose reconstruction following a nuclear reactor accident.

Citation: Bouville A, Linet MS, Hatch M, Mabuchi K, Simon SL. 2014. Guidelines for exposure assessment in health risk studies following a nuclear reactor accident. Environ Health Perspect 122:1–5; http://dx.doi.org/10.1289/ehp.1307120

## Introduction

Radiation exposure following nuclear accidents and emergencies is of great concern to the public and to the authorities tasked with emergency response and ensuring public health, safety, and well-being. Guidance has been published for many activities that are important after nuclear emergencies, accidents, and detonations [see Armed Forces Radiobiology Research Institute 2010; Becker 2004, 2005; Brodsky et al. 2004; Coleman et al. 2012; Department of Health and Human Services 2013; Goans and Wasalenko 2005; International Atomic Energy Agency 2002; National Council on Radiation Protection and Measurements (NCRP) 2005]. Most guidance focuses on medical planning, emergency response, and immediate consequence management but is limited for the collection of radiation exposure–related data needed to predict or estimate risks for late health effects. To address this important gap, we propose a set of guidelines to estimate radiation doses for evaluations of health risk.

The activities undertaken after the four major nuclear reactor accidents of the past are the best model we have for learning what needs to be improved to advance data collection for radiation exposure and dose assessment strategies. Here we discuss these accidents in the context of learning how to improve dose assessment for health risk studies (see National Academy of Sciences 1995; NCRP 2009; Simon et al. 2006b).

Because of concerns about potential health risks, two questions will likely emerge after any reactor accident:

Immediately after the accident: What adverse health effects should be expected as a consequence of the accident?
Years after the accident: What were the actual health consequences caused by the accident?

These questions call attention to two types of health risk evaluations that can be undertaken: risk projections and epidemiologic studies.

In risk projections the types and number of expected adverse effects resulting from an accident are predicted based on the estimated or assumed extent of human exposure and epidemiologic data from previous studies. Risk projections can be conducted well in advance of the expected occurrence of the health consequences (i.e., “early-phase” risk projections) or can be done many years after the radiation exposure (i.e., “late-phase” risk projections). A characteristic of risk projections is that they only require the estimation of average doses over population groups.

Epidemiologic studies are usually undertaken several years after the accident to allow time for the health consequences to be expressed, are based on the analysis of observed adverse health effects, and seek to ascertain risks of these adverse effects in comparison with the background or baseline rates. Such investigations typically involve the collection of exposure and outcome data for the study participants and require individual dose estimation.

The basic difference in these two types of studies is that risk projections generate expected rates of disease whereas epidemiologic studies generate observed rates of disease. In this commentary, we discuss dose assessment and data collection guidelines to support both types of studies.

Four past reactor accidents have each resulted in irreparable damages to the power plant and in substantial radiation exposures involving ≥ 1,000 people as a consequence of the releases of radioactive materials into the environment. The first of those accidents took place in 1957 at Windscale in the United Kingdom and was caused by a fire in the reactor, which was mainly used for the production of plutonium. The second accident took place at the Three Mile Island (TMI) power reactor in the United States in 1979 and was due to both mechanical and human errors. The third, and most severe, reactor accident was at the Chernobyl nuclear power plant in the former Soviet Union in 1986 and resulted from a series of human errors during the conduct of a reactor experiment. Finally, the Fukushima accident, which occurred in northern Japan in 2011, was the consequence of an earthquake-triggered tsunami that damaged the reactor cooling system.

The radiation health impacts of the Windscale, TMI, and Chernobyl accidents were projected or assessed on the basis of estimated radiation doses to the affected populations; similar efforts to estimate dose are in progress for the Fukushima accident. The activities conducted after each accident are briefly summarized below.

After the 1957 Windscale accident, the immediate concern was to evaluate the pathways of exposure to humans and to minimize exposure to radioactively contaminated foodstuffs. An early-phase risk projection was published 1 month after the accident (Arnold 2007). Late-phase risk projections were performed in the 1980s (Clarke 1990; Crick and Linsley 1982, 1984). No epidemiologic studies of exposure to the public were ever conducted.

Within a few months of the 1979 TMI accident, several early-phase risk projections estimated likely health effects to the population located ≤ 50 miles of the reactor site (Rogovin and Frampton 1980). Epidemiologic studies of cancer incidence and mortality were performed in the 1990s and in the early 2000s (Han et al. 2011; Hatch et al. 1990, 1997; Talbott et al. 2003; Wing et al. 1997).

Risk projections were published within a few years of the Chernobyl accident (Anspaugh et al. 1988; Ilyin et al. 1990; Parmentier and Nenot 1989). Extensive collaboration between the former Soviet Union and international organizations started in 1989 and resulted in a series of epidemiologic studies focusing mostly on thyroid diseases and, to a lesser extent, on childhood cancer (Cardis and Hatch 2011). Health effects resulting from the Chernobyl accident have been extensively reviewed [United Nations Scientific Committee on Atomic Radiation (UNSCEAR) 2011]; mental health appears to be one of the important public health problems arising from the accident (Bromet 2012).

Radiation-exposure data related to the assessment of doses received by the population affected by the Fukushima accident are still being collected. Early-phase dose assessment [World Health Organization (WHO) 2012] and risk projections have been published (Beyea et al. 2013; Ten Hoeve and Jacobson 2012; WHO 2013), and epidemiologic studies are being considered (Akiba 2012). Boice (2012) indicated that a study of mental disorders would be important because the population experienced a traumatic series of events, loved ones died, lives were disrupted, and concern over radiation remains widespread.

The approaches for estimating doses to representative populations near Windscale and TMI, or to individuals near Chernobyl, varied according to the type, quality, and amount of information that was available. Future accidents will also likely vary considerably on specifics of the accident and the exposure conditions. The general strategy to support dose estimation that we present here will accommodate many variations and includes

1. Identification of the target population
2. Collection of as many individual-based radiation measurements as possible for persons in the target population
3. Collection of individual personal and lifestyle information that can be used for the estimation of individual dose
4. Collection of information on the spatial and temporal patterns and variations of the radiation field
5. Calculation of realistic radiation doses with efforts to minimize sources of bias
6. Validation of the dose estimates by independent measurements or strategies
7. Qualitative and quantitative evaluation of the uncertainties associated with dose estimates.

These guidelines apply in large part to other types of nuclear accidents such as the Kyshtym accident in the former Soviet Union, which was caused by a chemical explosion of stored radioactive waste that occurred at the Mayak Production Association in 1957 (Akleyev and Lyubchansky 1994; Kossenko 1995; Kostyuchenko and Krestinina 1994), and to the detonation of nuclear devices such as those that were conducted in Nevada (USA) (Simon et al. 2006a), in the Marshall Islands (Simon et al. 2010b), and in Kazakhstan (Burkart 1996).

## Discussion

*Guideline no. 1: Create as complete a roster as possible of persons in the exposed area*. For long-term purposes, it is essential to compile as complete a list as possible of people exposed following a nuclear reactor accident. Although this will be difficult in the immediate aftermath, it needs to be accorded a high priority because the effectiveness of emergency/early response, and late-phase risk projections and epidemiologic studies, all depend to some extent upon having comprehensive population rosters with contact information.

The rosters should include all relevant members of the affected population, particularly those exposed to the full range of doses and subsets of the population who may potentially be more susceptible to adverse effects, including pregnant women and fetuses, children and adolescents, the elderly, and the infirm. Potential data sources that could be useful for identifying such individuals include population registers, hospital emergency and other departmental records, rosters obtained by response workers and volunteers, lists of students at local schools, business employees, and voter registration lists in the exposed geographic areas. In addition, public health outreach programs and media campaigns with the affected community may assist in identifying persons who should be on the rosters. Some of the strategies noted here have been discussed in more detail elsewhere (Centers for Disease Control and Prevention 2005, 2007).

*Guideline no. 2: Obtain as many individual-based radiation measurements as possible*. In this context, individual-based radiation measurements means measurements of radiation directly emitted from the body of exposed persons or from their excretions, secretions, or removed tissues. The ideal approach to a dose reconstruction for an epidemiologic study involves obtaining individual-based radiation measurements from all targeted participants of the epidemiologic study using well-calibrated measurement devices. This step is useful, but not essential, for a risk projection. Ideally, multiple measurements would be acquired for each individual.

For estimating external irradiation, biodosimetric assays can be useful: for example, electron paramagnetic resonance of tooth enamel or fingernails and assessment of radiation-related chromosomal damage, such as dicentrics measured within weeks to months after exposure, or reciprocal translocations in chromosomes of lymphocytes, assayed by fluorescence *in situ* hybridization up to many years after the exposure. Whenever feasible, we recommend collection and archiving of biological samples or teeth for eventual analysis.

For internal irradiation, there are two types of useful assays (International Commission on Radiation Units and Measurements 2002): *a*) direct bioassays, which measure radioactivity in the whole body or in specific organs using instruments external to the body such as whole body gamma counting or localized gamma spectrometry (e.g., lung counts after suspected inhalation of insoluble radionuclides), and *b*) indirect bioassays, which include measurements of radioactivity secreted or excreted from the body. The first class of assays can provide an estimate of the internal contamination and, thus, the dose rate from internal radioactivity. If the measurements are repeated over the entire time of dose delivery, the cumulative dose can be determined. The second class of assays can provide an indirect estimate of the integral dose until the time of the measurement, but such assays need to be supplemented with physiological biokinetic models and knowledge of the kinetics of the radionuclide intake.

The purpose of these measurements is to obtain data relevant to each exposed person so that the radiation dose specific to the person and to the organs of interest can be estimated with the greatest reliability and the least uncertainty. Generally speaking, the overall uncertainties in individual dose estimates are largest when no individual-based or environmental radiation measurements exist, smaller when there are radiation measurements in the environment, and smallest when radiation measurements are available for the exposed persons.

Individual-based radiation measurements are very helpful, even if not obtained for every exposed person, because these can be used to estimate doses to representative individuals or to validate the dose estimates obtained by other methods.

If individual-based measurements of exposed persons can be obtained, it may be possible, in theory, to reconstruct doses received, and their uncertainties, directly from the data. Within the framework of an epidemiologic study, this can be done if a criterion for study participant selection is the availability of an individual radiation measurement—as was the case for some of the Chernobyl studies (Stezhko et al. 2004). However, based on experience in many past health risk studies, it is unlikely that measurements would be available for all study participants due to practical limitations (Simon et al. 2010a), including *a*) competing needs for urgent medical care, *b*) insufficient numbers of high-quality instruments and of qualified personnel to use them for measuring large numbers of exposed persons, and *c*) the high cost of measurements.

Under most realistic scenarios, dose estimation is more complex than directly using instrument readings because dose estimates need to be supplemented with data on the environmental radiation field and foodstuffs as well as information on the ways in which persons were exposed. The five other guidelines apply to the common situation where the available individual-based radiation measurements are not sufficient to determine the radiation doses received by all study participants.

*Guideline no. 3: Collect, for each study participant, information on whereabouts at the time of exposure and relevant dietary data that can be used for the estimation of the radiation dose*. Detailed information on the manner in which each study participant was exposed is very important for reliable dose estimation. For risk projections, individuals are not identified but population groups are. For this type of assessment, the ideal approach for collecting information might consist of conducting interviews on representative members of each population group, stratified according to relevant factors such as age, sex, and ethnic group. For analytic epidemiologic studies, it would be ideal to collect individual information through administration of interviews to all study participants.

For assessing external exposure, the relevant questions relate to the whereabouts of the exposed person during the period of exposure, the type of building where the individual resided or worked, and the number of hours spent indoors each day at each location. For assessing internal irradiation, the relevant questions relate to the geographic origins of water and foods consumed, consumption rates, and preparation methods of potentially contaminated foodstuffs. Personal interviews should be conducted by means of a carefully prepared questionnaire that has been tested on a sample of exposed persons and is administered by trained personnel. When interviews of the exposed persons are not feasible due to young age or death, or participation rates are low, less ideal approaches can be used, including the use of proxy respondents (Chumak et al. 2008), focus groups (Schwerin et al. 2010), or official local or national records, which sometimes document the residential history of persons living in the area.

Interviews should be administered as soon as possible after the accident in order to maximize memory recall and the reliability of the responses, recognizing that confusion resulting from the trauma of the accident itself may limit the reliability of some interview data.

*Guideline no. 4: Collect as much information as possible on the spatial and temporal variations of the radiation field*. Spatial and temporal data on the radiation field can be used to make estimates of dose for risk projection studies by assuming representative lifestyles and diets. For epidemiologic studies, however, lifestyle and dietary data obtained in interviews need to be combined with environmental radiation measurement data to estimate the dose rates in air and the radionuclide intake rates at all locations and at all times where exposure took place.

For assessment of external exposure, it would be ideal to obtain accurate measurements of exposure rates of airborne radionuclides at all locations (both indoors and out) where the exposed persons spent time during the entire period of exposure. For assessment of internal exposure, the ideal approach would be in making measurements of the radionuclide concentrations in the air and in the foodstuffs that were consumed during the time of exposure. In general, measurements should begin as soon as possible after the accident, understanding that they may have to continue for weeks to years to properly characterize weathering and environmental losses of the radioactivity that, if unaccounted for, will lead to overestimates of doses (UNSCEAR 2011).

*Guideline no. 5: Calculate realistic radiation doses, minimizing sources of bias*. Radiation dose is usually not directly measured and, therefore, must be calculated or estimated from models that range from simple, for external exposure, to complex, for internal exposures. Unbiased and accurate dose estimates should be sought for all individual participants in epidemiologic studies and for population groups for risk projections. Developing unbiased estimates of dose implies that all pathways of exposure must be taken into account and that efforts be made to eliminate or minimize potential under- or overestimation in collected data and parameter values used in models.

To calculate doses for each participant in epidemiologic studies, relevant exposure information should be used first and supplemented, secondarily, with estimates from mathematical models as necessary. The amount and quality of available information will vary among participants and between studies, implying that the degree of reliance on models will also vary. An overarching principle is to give preference to data in this order: *a*) radiation exposure measurements on the participants themselves, *b*) radiation measurements on other people relevant to the study, *c*) environmental radiation measurements and, finally, *d*) mathematical models.

A common limitation for all health risk studies, regardless of whether they are risk projections or epidemiologic studies, is that some assumptions need to be made when performing the dose calculations. Assumptions should be carefully documented in order to meet the requirements for peer-reviewed publications and to enable modifications to the original assumptions in future analyses.

*Guideline no. 6: Validate the dose estimates*. The validation of the dose estimates is the process used to ensure that the dose estimates are as accurate as possible and do not reflect systematic biases. Because the use of unbiased dose estimates is critical to epidemiologic studies, it is important to perform as many validation tests as possible and to consider making adjustments to the dose estimation process as a result of those tests.

The ideal approach is to estimate the dose for a suitable proportion of the targeted population using a biologically related measure that correlates highly with dose and to compare the measurements made by the primary approach with estimates of doses made by other means (Simon et al. 2010a). As noted, there are bioassay techniques for validation of both external and internal exposure. Because of the overall uncertainties in the dose estimates as well as in the validation measurements, the validation process usually provides only an indication of substantial flaws in the primary measurement methods or parameter values (when models are used) used for dose reconstruction.

More difficult to validate are organ doses for which no assay exists, for example, the dose received from intakes of iodine-131. Here, the validation of the dose estimates can be performed using an alternative method such as model-based estimates of the atmospheric release, or an empirical approach [making use of environmental measurements of iodine-129 (a long-lived radioactive isotope)]. A validation approach based wholly on model calculations is discouraged if other alternatives exist because of the difficulties in choosing appropriate and unbiased model parameter values.

*Guideline no. 7: Evaluate dosimetric uncertainties*. Evaluating the uncertainties in dose estimates is important because it addresses questions of the reliability, accuracy, and overall validity of the dose estimates. This is mainly important for analytic epidemiologic studies and for late-phase risk projections. There are many sources of dosimetric uncertainty in environmental radiation measurements, the mathematical models and the parameter values used to supplement the gaps in the radiation measurements, lifestyle data-based personal interviews, and in the case of internal irradiation uncertainties in the metabolic and anatomic attributes of each person.

Dosimetric uncertainty has a number of characteristics that are relevant to making conclusions for both risk projections and epidemiologic studies. In particular, some measurements may pertain not to single persons, but to groups of people—for example, the average deposition of cesium-137 in a village. In such cases, not only is the parameter estimate shared among members of the group, but the uncertainty of the estimated parameter, including any bias that might be present, is also shared. As noted in Guideline no. 5, it is our recommendation that biases be minimized as much as possible. Because biases occur, by definition, with systematic or shared errors, sources of shared uncertainty need to be scrutinized carefully.

Although a single ideal approach to evaluate and account for all dosimetric uncertainties is not available, this is an area of active research (NCRP 2009). Until recent years, the evaluation of the uncertainties consisted of numerical simulations in which variability and lack-of-knowledge uncertainties were combined in Monte Carlo simulations. In that method, probability density distributions are assigned to the parameter values deemed to have a substantial influence on the dose estimate, and multiple realizations of individual doses are estimated (NCRP 1996). The primary limitation of many such simulations is that shared errors and intraindividual correlations are not accounted for. More sophisticated Monte Carlo procedures are now being developed to separate and distinguish between the shared and the unshared components. However, there are always concerns that all sources of uncertainty may not be taken into account. For example, there may be “unknown” exposure pathways or unsuspected relevant radionuclides, among other factors.

*Application of guidelines to two types of risk assessment.* These guidelines cover the activities to be conducted after a reactor accident and are important for the two types of risk assessment, the first of which we divide into two subcategories: *a*) early-phase risk projections conducted soon after the accident, that is, prior to the development of the health outcomes (cancers and other diseases), and *b*) late-phase risk projections carried out years after the accident. The second type of risk assessment employs epidemiologic studies to quantify the actual consequences of the accident on the basis of the observed adverse health effects. Table 1 summarizes the degree to which each of the seven guidelines applies to these two types of health risk evaluations.

**Table 1:** Value of applying guidelines to the different types of health risk studies.

Guideline	Strategy	Value to risk projection studies		Value to epidemiologic studies
		Early phase	Late phase	
1	Creation of a roster of exposed persons	Unnecessary	Unnecessary	Essential
2	Collection of individual-based radiation measurements	Useful	Useful	Highly useful
3	Collection of personal information on exposed persons	Unnecessary	Unnecessary	Highly useful
4	Collection of data on radiation field	Essential	Essential	Essential
5	Calculation of realistic unbiased doses	Useful	Essential	Essential
6	Validation of dose estimates	Unnecessary	Useful	Highly useful
7	Evaluation of uncertainties of dose estimates	Unnecessary	Highly useful	Highly useful

The usual purpose of early-phase risk projections is to develop plans for remediation, recovery, and treatment of health conditions. For early-phase risk projections, the most important activity consists of collecting data on the radiation field (Guideline no. 4) so that unbiased doses can be calculated (no. 5) for representative but unspecified members of the exposed population. In so doing, individual-based radiation measurements (no. 2) are useful, but not essential. The creation of a roster of exposed persons (no. 1), the collection of personal information on exposed persons (no. 3), the validation of the dose estimates (no. 6), and the evaluation of uncertainties in those dose estimates (no. 7) are not necessary for early-phase risk projections.

In contrast, the usual purpose of late-phase risk projections is to replace an epidemiologic study that cannot be conducted for various reasons including difficulty in assembling a cohort, attrition of exposed study participants without good documentation of causes of death, or infeasible costs. For late-phase risk projections, only Guideline nos. 4 and 5 are essential. However, for late-phase risk projections that strive for high reliability and that may be considered as substitutes for epidemiologic studies, all guidelines but nos. 1 and 3 will substantially improve their validity.

For epidemiologic studies, all seven guidelines are important, but three are essential: the creation of a roster of exposed persons (no. 1), the collection of radiation field measurements (no. 4), and the calculation of realistic unbiased doses for all study participants (no. 5). The latter activity must be carried out using as many individual-based radiation measurements (no. 2) and personal interviews (no. 3) as possible. The dose estimates obtained should be justified by means of a validation process (no. 6). The evaluation of the uncertainties in the dose estimates (no. 7) also is important and “highly useful,” with the caveat that, because a great deal of subjectivity is involved in the evaluation process, the uncertainty results may vary substantially from one group of experts to another.

Finally, it is important to emphasize that the conduct of epidemiologic studies requires the involvement and consent of the participants and, by extension, effective methods of risk communication with the affected community. Results of risk-projection studies, which are carried out without formal contact with the exposed populations, need to be explained in detail using nontechnical terms to prevent misinterpretation.

## Conclusions

In this commentary, we present seven guidelines for what we consider to be an ideal approach for collecting data to be used for estimating radiation doses to the affected populations following a nuclear reactor accident. Implementing a subset of these guidelines will facilitate development of unbiased projections of future risk of cancer or other adverse health outcomes. Implementation of all seven of the guidelines is optimal for determining the true health consequences from the accident. The practical limitations to the proposed approach are typically competing needs for emergency care and other special circumstances that would accompany any accident and that might preclude comprehensive collection of some relevant data, as well as insufficient availability of equipment, personnel, and financial resources.

These seven guidelines should be carefully considered at the beginning stage of recovery from a reactor accident. All efforts to estimate future risks or to characterize actual disease incidence in the exposed population will benefit from implementing well-planned data collection strategies.
